# Entomopathogenic Fungi and Bacteria in a Veterinary Perspective

**DOI:** 10.3390/biology10060479

**Published:** 2021-05-28

**Authors:** Valentina Virginia Ebani, Francesca Mancianti

**Affiliations:** 1 Department of Veterinary Sciences, University of Pisa, viale delle Piagge 2, 56124 Pisa, Italy; francesca.mancianti@unipi.it; 2 Interdepartmental Research Center “Nutraceuticals and Food for Health”, University of Pisa, via del Borghetto 80, 56124 Pisa, Italy

**Keywords:** acari, acaripathogens, bacteria, entomopathogens, fungi, mites, ticks

## Abstract

**Simple Summary:**

Several fungal species are well suited to control arthropods, being able to cause epizootic infection among them and most of them infect their host by direct penetration through the arthropod’s tegument. Most of organisms are related to the biological control of crop pests, but, more recently, have been applied to combat some livestock ectoparasites. Among the entomopathogenic bacteria, *Bacillus thuringiensis*, innocuous for humans, animals, and plants and isolated from different environments, showed the most relevant activity against arthropods. Its entomopathogenic property is related to the production of highly biodegradable proteins. Entomopathogenic fungi and bacteria are usually employed against agricultural pests, and some studies have focused on their use to control animal arthropods. However, risks of infections in animals and humans are possible; thus, further studies about their activity are necessary.

**Abstract:**

The present study aimed to review the papers dealing with the biological activity of fungi and bacteria against some mites and ticks of veterinary interest. In particular, the attention was turned to the research regarding acarid species, *Dermanyssus gallinae* and *Psoroptes* sp., which are the cause of severe threat in farm animals and, regarding ticks, also pets. Their impact on animal and human health has been stressed, examining the weaknesses and strengths of conventional treatments. *Bacillus thuringiensis*, *Beauveria bassiana* and *Metarhizium anisopliae* are the most widely employed agents. Their activities have been reviewed, considering the feasibility of an in-field application and the effectiveness of the administration alone or combined with conventional and alternative drugs is reported.

## 1. Introduction

Biological control has been defined as ‘‘the intentional introduction of an exotic biological agent for permanent establishment and long-term pest control’’ [1].

The present study aimed to review the papers dealing with the biological activity of fungi and bacteria against some mites and ticks of veterinary interest. In particular, attention was turned to the research regarding acarid species, *Dermanyssus gallinae* and *Psoroptes* sp., which cause severe threat in farm animals and, regarding ticks, also pets. Furthermore, some agents can involve human health, too.

## 2. Entomopathogenic Fungi

Several fungal species are well suited to control arthropods, being able to cause epizootic infection and most of them infect their host by direct penetration through the arthropod’s tegument [2]. Most of the organisms are related to biological control of crop pests, but, more recently, have been applied to combat some livestock ectoparasites.

Acaripathogenic fungi have been reviewed by Chandler et al. [3] and classified as follows:

(a) Acari-specific pathogens, important regulators of mostly phytopathogenic mites (i.e., *Hirsutella* sp.). Different species within the genus *Hirsutella* (anamorphic status of *Ophiocordyceps*) have been reported as able to infect acari or insects also [3]. However, they exhibit a narrow range of hosts, acting very differently regarding non-specialist fungi, such as *Beauveria* and *Metarhizium*, which have more than 700 hosts [4]. Other specialist entomopathogenic fungal genera are zygomycetes, belonging to the order *Entomophtorales* such as *Neozygites* and *Conidiobolus*. As the other Zygomycota, these molds develop broad hyaline coenocytic hyphae [5], producing sporangiospores and, being homothallic, zygospores, too.

(b) Non-specific fungal species (infecting both acari and insects) are the most widely studied. The main genera *Beauveria*, *Metarhizium*, *Paecilomyces* and *Verticillium*, although not completely ecomorphologically adapted to the life cycle of specific arthropods [6], contain acaripathogenic species such as *Metarhizium anisopliae, Beauveria bassiana, Beauveria brongniartii, Verticillium lecanii, Paecilomyces eriophyes, Paecilomyces farinosus, Paecilomyces fumosoroseus* and *Paecilomyces terricola.*

(c) Minor species, not deeply studied, rarely found as pathogens, and not studied for biological control purposes are a range of fungi (*Aspergillus fumigatus, Penicillium insectivorum, Trichothecium roseum*) occasionally isolated from tick/mite cadavers.

Entomopathogenic fungi (EPFs) have been identified by their growth onto insect cadavers and can be commercially produced to act as biopesticides. Species of *Beauveria, Metarhizium, Lecanicillium* and *Isaria* are relatively easy to mass produce [7]. One of the main concerns about their extensive employ would be related to their sensitivity to temperature as well as ultraviolet radiation [8] and to the presence of a suitable moisture degree, to allow the conidia to germinate [9]. On the other hand, EPFs seem to have a negligible risk of inducing resistance [10], despite their long-term persistence in the environment.

*B. bassiana* (common name “white muscardine fungus”, teleomorph *Cordyceps bassiana*) is a cosmopolitan, soilborne ascomycete, acting as a facultative necrotrophic arthropod-pathogenic [11], occurring as saprotroph and plant endophyte. Fungal conidia are able to attach, produce hyphae and penetrate the arthropod body, utilizing it for their development [12]. After invading the hosts’ body, fungal mycelium propagates in hemolymph. After the host’s death, mold goes on with a saprophytic growth on the cadaver, producing conidia for new dispersal and infection cycles [13]. In a view of a large-scale application of *B. bassiana* for pest control, several studies about the resistance mechanism to physical factors are ongoing [14,15,16].

Moreover, *B. bassiana* can produce beauvericin, a secondary metabolite capable of increasing oxidative stress leading to cell apoptosis [17].

This indirect action has recently been studied against all stages of *Sarcoptes scabiei* [18].

*M. anisopliae* (common name “green muscardine fungus”) is considered as a complex of species, morphologically quite similar of soilborne ascomycetes, widely used for the biological control of several arthropod pests. The species has been revised by Bischoff et al. [19], while the whole genus has been recently revisited [20].

The species cited as mycoacaricide in the present study were *M. anisopliae, Metarhizium robertsii, Metarhizium brunneum* (*M. anisopliae* complex) and *Metarhizium flavoviride* and *Metarhizium pemphigi* (*M.flavoviride complex*).

## 3. Entomopathogenic Bacteria

Among the entomopathogenic bacteria (EPBs), *Bacillus thuringiensis* showed the most relevant activity against arthropods.

*B. thuringiensis* is a Gram-positive, rod-shaped, spore-forming bacterium, innocuous for humans, animals and plants. It can be isolated from different environments, such as soil, rhizosphere, phylloplane, freshwater, and grain dusts; furthermore, it can be found in invertebrates and insectivorous mammals [21].

Its entomopathogenic property is related to the production of highly biodegradable proteins. Its action to the insect pest relies on insecticidal toxin and an array of virulence factors [22]. *B. thuringiensis* produces, upon sporulation, insecticidal crystal inclusion formed by several proteins named Cry or Cyt proteins. These proteins have been proven to be toxic to insects belonging to the orders *Lepidoptera, Dipteran, Coleoptera, Hymenoptera, Homoptera, Orthoptera* and *Mallophage* [22].

Furthermore, the entomopathogenic activity of *B. thuringiensis* is related to other virulence factors, including exotoxins and extracellular proteases. Exotoxins are heat-stable water-soluble and low-molecular-mass compounds (701 Da), highly toxic to a wide range of insect species by the oral route [23,24]. Different extracellular proteases, such as serine protease, chitinase, collagenase, have been identified [22,25].

It has been observed that virulence factors are able to breach the epithelial cells of the insect midgut and increase the insecticidal activity of Cry protein. Moreover, virulence factors can protect *B. thuringiensis* from the innate immune system through the cleavage of antimicrobial peptides, whereby the insecticidal activity of the Cry protein is enhanced [21].

*B. thuringiensis*, because of its known entomopathogenic activity, has been used worldwide for biological control against several agriculture pests for a long time.

Nowadays, in fact, commercially available products based on crystals and/or spores from environmental strains of *B. thuringiensis*, as well as trans-conjugant and recombinant strains, are used for the population control of different arthropod groups, including Lepidoptera (mainly *B. thuringiensis* var. *kurstaki, thuringiensis* or *aizawai*), Diptera (*B. thuringiensis* var. *israelensis*), and Coleoptera (*B. thuringiensis* var. *tenebrionis* and *san diego*) [26]. More recently, it has been proposed as an agent against parasites of human and veterinary concern, too.

Besides *B. thuringiensis*, *Lysinibacillus* (formely *Bacillus*) *sphaericus* is employed for preventing and controlling pests. Both agents are the only commercial entomopathogenic bacteria that are produced using mass production techniques and sold in sufficient commercial quantities. *L. sphaericus* is commonly isolated from soil and aquatic habitats. At the end of its vegetative life cycle, it produces round spores in a swollen “club-like” terminal or subterminal sporangium. Moreover, *L. sphaericus* can produce an intracellular protein toxin (SSII-1) and a parasporal crystalline toxin at the time of sporulation. Its mosquitocidal activity has been demonstrated mainly with Culex mosquitoes, followed by *Anopheles*, *Mansonia*, and some *Aedes* spp. [27,28].

Other members of the genus *Bacillus* have shown entomopathogenic properties. Among them, the most commonly employed against agricultural pests is *Brevibacillus* (formerly *Bacillus*) *laterosporus.*

This is an aerobic, spore-forming bacterium which was originally isolated from water [29,30]. It produces a canoe-shaped parasporal body which cradles the spore and is firmly attached to it. Since McCray [31] isolated *B. laterosporus* from diseased bees in 1917, it has been supposed that this bacterium might be an insect pathogen. However, *B. laterosporus* is recognized to be a saprophyte living on the dead remains of bee larvae and it is not always present in these insects [32,33].

Successively, *B. laterosporus* demonstrated pathogenic activity against black fly larvae *Simulium vittatum* [33]. Black flies are important nuisance pests of humans and farm animals, as well as being vectors of arboviruses and river blindness, caused by the nematode *Onchocerca volvulus* [34]. The entomopathogenic activity of *B. laterosporus* was also proven against larvae of the mosquito *Culex quinquefasciatus* and *Aedes aegypti* [33,35,36,37] and houseflies *Musca domestica* [38].

Several studies have been carried out to verify the role of endosymbiont bacteria as possible entomopathogenic agents. Endosymbionts are intracellular obligate bacteria that contribute to the fitness of the tick including, nutrient provision and host defense; in some cases, it has been supposed that they can cause phenotypic and reproductive alterations in their arthropod hosts [39].

Several studies in fact observed anomalies of parthenogenesis [40,41], reproductive incompatibilities between infected and uninfected individuals [42], and the disturbance of oogenesis [40]. These reproductive alterations may cause the mortality of male embryos [43] and give rise to populations consisting only of haploid individuals [44].

The most encountered species belong to genera *Spiroplasma*, *Cardinium*, *Schineria*, *Rickettsiella*, *Wolbachia* [39,45].

*Spiroplasma* sp. are bacteria responsible for sexual determination in insects. Tinsley and Majerus [46] demonstrated that *Spiroplasma* sp. are male-killing bacteria causing a female-biased offspring ratio in female ladybirds *Anisosticta novemdecimpunctata*. Although *Spiroplasma* sp. are usually considered to be pathogens, they have also been reported to be symbionts in some insects and the potential role of mosquito spiroplasmas as vector control agents has been discussed [47].

Bacteria of the genus *Cardinium* have been associated to the parthenogenesis of parasitoid wasps and recognized as a symbiont of the phytophagous mite *Tetranychus pueraricola* [41,48].

*Schineria* sp. bacteria have been previously isolated from the larvae of *Wohlfahrtia magnifica* (Diptera: Sarcophagidae), a myiasis-causing fly species for most domestic animals [49,50]. Toth and colleagues [50] suggested that *Schineria* has a strong chitinase activity and may contribute to the development of fly larvae and influence the metamorphosis of *W. magnifica*.

*Rickettsiella* spp. are Gram-negative, obligate intracellular bacteria of the family Coxiellaceae. Currently, the genus comprises three widely recognized entomopathogenic species, and their pathotypes, *Rickettsiella popilliae*, *Rickettsiella grylli*, and *Rickettsiella chironomi* [51,52].

All species are highly fastidious intracellular pathogens and typically target the fat body and hemolymph cells of the host. The infective cells are typically small, dense rods ingested during feeding which traverse the midgut epithelium and enter the hemocoel, where they gain entry to host cells through endocytosis. Once within the cell, pleiomorphic forms develop within the cytoplasmic vacuoles, varying from bacteria-like secondary cells to large, round rickettsogenic stroma. As the disease develops, characteristic protein crystals form and cells revert to small rickettsia. Eventually, infected cells undergo lysis, releasing masses of rickettsia and crystals into the hemolymph.

Concern has been raised over potential inflammation and infection induced by entomopathogenic *Rickettsiella* in vertebrates [53], so care should be taken when working with these organisms [54].

*Wolbachia pipientis* was first detected in the common household species *Culex pipiens* by Hertig and Wolbach in 1924 [55]. This is the most widespread bacterial endosymbiont infecting terrestrial arthropods, mainly insects, but some arachnids, freshwater crustaceans, and filarial nematodes too [56]. It has been observed that *Wolbachia* may confer in infected Diptera species some resistance versus insect pathogens [57].

Different mosquito trials demonstrated that *Wolbachia* must be carefully assessed for use as a biological control agent; however, the effect depends on the mosquito species investigated, as well as on the *Wolbachia* strain [58].

Cytoplasmic incompatibility has been reported to occur in insects and arthropods infected with *Wolbachia* sp. [59,60,61].

This characteristic is a reproductive incompatibility between infected males and females that are either uninfected or infected with different strains of the endosymbiont [60,62]. The symbiotic bacteria spread in an arthropod population, causing a reduction in fitness through failed mating [63].

It has been supposed that the presence of *Wolbachia* may have direct consequences on the development of other pathogens in the same arthropod vector, and also indirect effects on the epidemiology of pathogens through impacts on the dynamics and genetic diversity of the vector [64,65].

## 4. Ticks

Ticks are large-bodied bloodsucking, nonpermanent parasitic Acari, feeding exclusively on vertebrates. They are divided into three families, among which Ixodidae (hard ticks) represent an important concern for mammalian health, although some of them also feed on birds, and can be carried between continents. The life cycle includes eggs, one larva and one nymphal instar, adult male and female. Life cycles are classified based on the number of times the stages change hosts. The ticks start to feed as larvae, then as nymphs, and finally as adults, even if, in some species, males do not ingest blood. Each generation may be 1 or 2 years, although some species may take 3 to 6 years [66].

Most Ixodid species change three hosts, and molts of juvenile stages occur on the ground. A few of them are referred to as one-host ticks, spending most of their life on a unique host and dropping to the ground for oviposition (i. e. *Rhipicephalus microplus*). Two-host ticks feed on the first host, molt in nymph and feed again. Engorged nymphs fall to ground and molt into adults that feed onto the second host, mate, then females drop and lay thousands of eggs, which are left among the decaying vegetation at protected sites, where a high relative humidity will ensure their survival [67]. Ticks can be more (*R. microplus*) or less (*Ixodes ricinus*) host specific, depending on their species [66].

There has been a shift of ticks to elevated latitudes and altitudes, would be due to climate change, along with the host abundance [68,69,70], so the area of distribution of some ixodid species has expanded in the last few years. Among the causes of tick introduction and spread, the uncontrolled movements of domestic or wild animals, climate trends, and changes in the use of land resources that allow hosts to increase have been reported. Ticks introduced into a region where there is no competition with other species of ticks, are, in fact, able to colonize the complete range of abiotic conditions compatible with their biology [71]. However, the effects from human activities appear more important in modifying biotopes, influencing the infection by pathogens, of ticks [67].

Ticks exert a direct damage, feeding on their host. Saliva and/or mouthpart penetration can induce a toxic reaction in hosts, such as tick paralysis [72,73], or allergic state in human patients [74]. Heavy tick infestation can cause severe anemia, considering that an adult female tick can feed up to 2.0 mL of blood from the vertebrate host [75]. Conversely, many tick species have a role in the transmission of several pathogens, zoonotic too, causing an indirect damage. Pathogens transmitted by ticks include the greater part of the agents of vector-borne diseases in temperate areas, with a public health impact mostly unquantified [76]. Tick-borne diseases affect about the 80% of the world’s cattle population, with a strong economic impact, mostly in developing countries [71].

As obligate hematophagous ectoparasites, ticks can ingest a huge amount of blood (up to 100-fold their body weight) [77], so they can easily transmit bacteria such as *Anaplasma*, *Ehrlichia*, *Borrelia* and *Coxiella.* Piroplasms (*Babesia* and *Theileria*), *Cytauxzoon* and *Hepatozoon* spp. complete their life cycle in hard ticks. One hundred and sixty tick-borne viruses are known, among which are tick-borne encephalitis and Crimean Congo hemorrhagic fever [70].

The current conventional acaricide treatment consists in the administration of different chemicals. The most employed drug classes are synthetic pyrethroids, organophosphates, amitraz, fipronil, insect growth regulators, macrocyclic lactones and isoxazoline. The compounds can be administered systemically or by direct application on the coat, alone or in mixture, with differences among the countries. Moreover, in companion animals, isoxazoline is administered per os. Anyway, the massive administration of acaricide drugs has made a resistant tick population [78]. To the best of our knowledge, the first report of the acaricide (organochlorine) resistance of cattle tick dates back to the half of the past century [79]. Acaricide resistance increased after the development of other compounds and will take place after exposure to any new molecules.

*R. microplus* (cattle tick) is one of the most studied Ixodidae, regarding to its acaricide resistance. Being a one-host tick, it spends most all its life cycle on the same host. Therefore, it is exposed to acaricide longer and has become resistant worldwide to almost all classes of chemicals. These ticks are reported as resistant to acaricide drugs [80,81,82,83,84,85,86,87,88,89,90,91] and multidrug resistant strains have been reported, also [92,93].

*R. sanguineus* (brown dog tick), conversely, is a three-host tick, and dogs are the primary host species, although infection of different animal species can occur in certain areas [94]. It can infest homes and is able to complete its life cycle indoors [95]. Acaricide resistance largely occurs in this species, too [96,97,98,99].

The rise of acaricide resistance in ticks is a serious concern, whose real extent is unknown [94]. However, several studies dealing with the mechanisms of resistance have been accomplished in the last few years [100,101,102,103,104], indicating that such resistance is due to genetic changes of tick populations, whose mechanisms would be linked to modifications to the target site, metabolism of compound alterations, or a decrease in the ability of the drug to cross the outer protective layers of the tick’s cuticle [105].

### 4.1. Fungi

Natural infections caused by *Aspergillus flavus*, *A. fumigatus* and *P. insectivorum* in all stages (mostly adults) and eggs of examined ticks have been described since 1964 [106]. Afterwards, 17 different fungal species were reported to occur in diseased *I. ricinus*, *Dermacentor marginatus* and *D. reticulatus*. Engorged females of *I. ricinus* appeared more prone to fungal infection in summer seasons [107]. The most pathogenic molds were *A. parasiticus, B. bassiana, Beauveria tenella, V. lecanii* and *P. fumosoroseus.* Similarly, *B. bassiana, B. brogniarti, P. farinosus, P. fumosoroseus, V. lecanii* and *Verticillium aranearum* was able to colonize *I. ricinus* free in the environment from Denmark [108], mostly being engorged females in autumn, suggesting a possible activity of EPFs as regulators of these populations. *A. ochraceus*, *Curvularia lunata*, and *Rhizopus arrhizus* were isolated from naturally infected *R. sanguineus* and were found able to kill them [109,110]. Recently, *A.parasiticus*, along with *Penicillium steckii* and *Scopulariopsis brevicaulis*, was found to contaminate a laboratory-reared colony of *I. ricinus* [111].

The control of ticks by entomopathogenic fungi has been widely studied and, differently from insects, tick eggs are sensitive [112]. Tick species differ in their behavior, range of hosts and life cycle, so also, their sensitivity in comparison to a fungal species is not the same [113]. Furthermore, ticks are reported to be more tolerant to EPFs than other arthropods, so the amount of the inocula for tick control purposes should be larger. Different stages of ticks would exhibit differences in sensitivity versus EPFs. *R. sanguineus* engorged females and unfed other stages appeared more prone to fungal infection with *M. anisopliae* and *M. flavoviride* [114]. Nymphs were reported as less sensitive when compared with other stages [113,115]. A slight difference of sensitivity to *M. brunneum*, between adults and nymphs of *I. scapularis* was also reported [116,117] and larvae are considered the most susceptible stage to EPFs [118].

*Metarhizium* and *Beauveria*, when cultured in a liquid medium, can produce yeast-like propagules, known as blastospores. These fungal stages have also been checked for their entomopathogenic action, being able to easily penetrate cuticles [119].

EPFs recognize their target host, then conidia adhere and germinate on its cuticle, developing hyphae and appressoria. Such fungal structures exert a mechanical pressure along with enzyme secretion, allowing the fungi to cross the cuticle, invade the host’s whole body, causing the death and colonizing the cadaver with their mycelium, emerging from the cuticle to continue the vegetative cycle.

EPFs, to start the host invasion, must overcome the epicuticle (outer layer of cuticle), mostly composed by esterified lipids, different among the hosts’ species [120,121]. The ability to colonize more host species based on the recognition of such lipids makes the fungi specialist or generalist. Furthermore, the adherence of conidia seems to be mediated by several proteins and lipolytic enzymes [122]. Lipolytic enzymes secreted by EPFs also seem to cause alterations in the lipid balance of ticks, hampering their survival and decreasing their reproductive capacity [123,124].

The pattern of invasion of *M. anisopliae* in ticks is characterized by a simultaneous internal and cuticular fungal growth, unlike insect colonization by the same mold. This can be also due to the different composition of the cuticle between insects and ticks. The latter have a lower proportion of chitin and differences in the binding of proteins in female alloscutum (to allow a rapid expansion during engorgement), so this different composition makes ticks more prone to fungal attacks. This rapid and extensive cuticle degradations would hasten the tick death, following the water loss [125]. Furthermore, a serious cytotoxic impact of *M. robertsii* on *R. microplus* hemocytes has been recently reported [126,127].

*M. anisopliae* is the most studied fungal species. Its most prominent features on *R. microplus* have been recently reviewed [128]. Striking differences of entomopathogenic activity among the isolates have been reported [129], probably due to genetic differences [128]. Furthermore, a trial on cattle did not yield the same results as in-field studies [130]. The mold acted as active, along with *B. bassiana* [131] as well as a blastospore suspension, together with *B. bassiana* and *M. robertsii* [132]. *R. microplus* was sensitive to commercial conidial suspensions in vegetable oils [133] and both in vegetal and mineral oils, applied on the grass, killing 100% of ticks and lasting in the environment up to 60 days [134]. A similar good efficacy against *R. microplus* was reported for a formulation containing both microsclerotia and blastospores of *M. robertsii* [135].

*M. anisopliae* appeared active against *Rhipicephalus variegatus*, *R. sanguineus* and *Ixodes scapularis*, but led to a limited mortality in *Dermacentor variabilis*, which was sensitive to *B. bassiana* [116]. It was proven to be effective against *Amblyomma parvum* [136] and *Haemaphysalis qinghaiensis*, such as *B. bassiana* [137]. In a trial on *Dermacentor albipictus* larvae, a spray application of *M. anisopliae* was more effective and active in a shorter time, such as *M. brunneum*, when compared to *B. bassiana* [138].

*M. anisopliae* appeared more active than *B. bassiana* against *I. ricinus* and to a lesser extent against *D. reticulatus* [139]. Strains of *M. anisopliae* and *B. bassiana* were able to kill engorged females of *Hyalomma anatolicum*, yielding better results in comparison with *Paecilomyces lilacinus* [140], while strains of *M. anisopliae* and *P. lilacinus* were more pathogenic than *B. bassiana* against *R. microplus* [129]. Similarly, *M. anisopliae* was reported to be very pathogenic against *Haemaphysalis longicornis*, unlike *B. bassiana* [141], while this latter fungal species was reported to be very effective against the same tick in another study [142]. These remarks would indicate huge differences among fungal populations and the usefulness of testing fungi isolated from the same environment where selected ticks occur. *M. brunneum* was successfully administrated to *I. scapularis* in a granular formulation [117,143]. This fungal species showed marked differences in activity against *Rhipicephalus annulatus* under field conditions [144].

*B. bassiana* was assayed on *R. microplus* in vitro and in vivo on affected cattle, with very promising results [145,146], as well as on *Hyalomma lusitanicum* both in vitro and on field, applied inside wild rabbit burrows [147,148]. The mold was active against unfed adults of *Amblyomma americanum* [149], as well as against amitraz-resistant and amitraz-sensitive *Rhipicephalus decoloratus*, without detecting any significant difference [150].

*M. anisopliae* was reported to inhibit 92% *Rhipicephalus appendiculatus*, while *B. bassiana* inhibited 80% [151]. Furthermore, a synergistic effect of both EPFs on *Rhipicephalus appendiculatus* and *Amblyomma variegatum* [152,153] was referred, too. In a recent comparative study on the efficacy of the autodispersion of commercially available strains of *M. anisopliae* and *B. bassiana* against nymphal stages of *R. sanguineus*, the latter fungal species was faster in killing ticks and more efficient in sporulating on cadavers, allowing a mold dissemination [154].

However, strong differences in sensitivity to *M. anisopliae* and *B. bassiana* among different populations of *R. microplus* are reported [155,156,157,158,159,160]. For these reasons, preliminary in vitro assays of different fungal strains against the selected tick population are mandatory, also considering the sublethal effects on reproduction, then on future population size in the environment [161].

*Isaria fumosorosea*, *Isaria farinosa* and *Purpureocillium lilacinum* (formerly included within the genus *Paecylomyces*) are considered as important EPFs and scored active against *R. microplus*, acting also in reducing the egg production from treated adult females. *I. fumosorosea* was able to reduce the hatching percentage of treated eggs but was scarcely effective on larvae [162]. *I. fumosorosea* induced a low mortality of larvae in *R. sanguineus* [114], in *D. reticulatus* and *I. ricinus* [136]. For these reasons, its use as mycoacaricide against ticks is not recommended [139].

*S. brevicaulis* was recovered from *D. variabilis* [163] and, although this genus is considered as a minor EPF [164,165], it was assessed as capable of protecting this tick species from the desiccation induced by *M. anisopliae* [166]. It is currently considered as a pathobiont, transstadially transmitted in winter ticks, able to kill experimentally infected ticks, but without significantly affecting eggs and larvae [167].

The activity of EPFs can be affected by environmental conditions (high temperature, desiccation, strong solar radiation) [113]. For these reasons, the formulation is essential. Oily formulations are effective in protecting conidia from solar UUVV irradiation and from the loss of humidity [128], and calcium alginate beads with granular corn starch or chitin powder as nutrients were able to protect *M. pemphigi* blastospores encapsulated from drying [168].

To avoid a waste of inoculum, small areas are most suitable for testing. The results of pen trials yielded variable results [161]. Liquid or solid formulations can be applied with fertilizer spreaders [169] and the mycoacaricide can be applied on pastures by aerial spraying [170]. EPFs can be directly applied on the host, but they must be able to overcome several barriers such as skin temperature, pH, sebum and sweat [161].

Lastly, the effectiveness of an integrated control has been reported. The mycoacaricide activity was enhanced, when fungal stages were added with deltamethrin to control pyrethroid-resistant *R. microplus* [171,172] or with cypermethrin and chlopyriphos [173]. Similarly, *I. scapularis* was sensitive to fipronil, added to *M. anisopliae* [174,175], as well as *R. sanguineus* to *M. anisopliae* plus cypermethrin [176]. An integrated alternative control of *R. microplus* by both essential oils and entomopathogenic fungi (*M. anisopliae* and *B. bassiana*) indicated the suitability of *Pelargonium graveolens* essential oil that is not able to inhibit these EPFs and, when *B. bassiana* is not involved, also *Lavandula hybrida* [177]. Similarly, an integrated control of *I. scapularis*, was achieved with a synthetic pyrethroid acaricide, *M. anisopliae* strain F52 and a mixture of essential oils [178].

Other alternative methods of tick control are the use of entomopathogenic nematodes alone [179] or in oily emulsions [180], plant-derived compounds [181,182,183] or vaccines. Anti-tick vaccine Bm86 is commercially available [184], mostly active on *R. annulatus* and to a lesser extent on *R. microplus* [185]. EPFs active against different tick species are summarized in Table 1.

### 4.2. Bacteria

Some bacterial species have been demonstrated to be pathogenic for ticks; thus, they are considered useful for biological control. Among EPBs, *B. thuringiensis* is the most studied agent with activity against ticks [195] and is largely employed in commercial insecticide formulations. The pathogenic action of *B. thuringiensis* normally occurs after the ingestion of spores by ticks, and the crystalline inclusions containing insecticidal δ-endotoxins specifically interact with receptors in the insect midgut epithelial cells [196].

Studies about the effectiveness of *B. thuringiensis* against ticks showed that this property is strongly related to tick species and different tick developmental stages [195,197,198]. In vitro investigations reported the activity of *B. thuringiensis* against *Hyalomma dromedarii*, *Argas (persicargas) persicus*, and *R. microplus*, *I. scapularis*, *I. ricinus*, *D. reticulatus* [199].

Szcsepanska and coworkers [199] tested four environmental strains of *B. thuringiensis* and one commercially available product (Vectobac) containing *B. thuringienis* against ticks of the species *I. ricinus* and *D. reticulatus*. Vectobac was not active against both tick species, whereas two environmental *B. thuringiensis* strains proved to be efficient against *I. ricinus* and *D. reticulatus*, with the mortality rate for ticks assessed as being up to 80%. Moreover, *D. reticulatus* males were the most sensitive ticks to bacteria. The authors found similarity between the most and least efficient *B. thuringiensis* strains in enzymatic profiles (lipases, phosphatases, proteases, and chitinases), and for this reason they supposed that the detected enzymes have a limited role in the pathogenicity profile of the bacterial strains against ticks.

The effectiveness of *B. thuringiensis* against other tick species was also observed in further investigations. The pathogenicity of *B. thuringiensis* variety *kurstaki* was tested against the black-legged tick *I. scapularis*. *B. thuringiensis* was active against engorged larvae with LC50 of 107 spores/mL [198].

The sensitivity of the soft ticks *A. persicus* and the hard ticks *H. dromedarii* versus the commercial product Vectobac was assayed by Hassanain et al. [197] and mortality rates over 70% were observed. These results are not fully in accordance with those found by Habeeb and El-Hag [200] that did not record mortality rates after dipping *H. dromedarii* in Vectobac, but when they injected this commercial insecticide to *H. dromedarii* hemocoel, increased mortality rates in the next 48 h were observed.

In Mexico, four strains of *B. thuringiensis*, among 60 tested native strains, caused mortality rates exceeding 90% of adult *R. microplus* on the 20th day of the immersion test assay [195].

To the best of our knowledge, studies about the sensitivity of *R. sanguineus* to *B. thuringiensis* are not present in the literature. However, Renè-Martellet et al. [201] studied the microbiota composition of *R. sanguineus* ticks collected in different geographical areas (Senegal, France, Arizona) and found that each bacterial microbiota was dominated by three genera: *Coxiella*, *Rickettsia* and *Bacillus*. In particular, *Rickettsia* and *Coxiella* were the two main genera detected in females, whereas males had a higher proportion of *Bacillus*; however, the nature of the association between male *R. sanguineus* ticks and *Bacillus* spp. was not characterized.

The tick pathogenic property of *Proteus mirabilis* has been observed by Brown et al. [202] in a laboratory population of *D. andersoni*. In fact, a high rate of mortality was observed among all developmental stages of engorged ticks from which *P. mirabilis* was cultured. Mortality was preceded by disease in ticks, that had discoloration caused by the release of black decayed blood into the hemocoel, when the gut decomposed. At death, the cuticle was badly decomposed and was easily ruptured. Furthermore, the viscous fluid in the body cavity had a characteristic putrefactive odor.

Even though these findings suggest a relevant activity of *P. mirabilis* as entomopathogen, its use against ticks is not recommended because this is an opportunistic bacterium able to cause infections in humans and animals [203].

Among endosymbiont bacteria, *W. pipientis* is the species most frequently found in ticks. It has been detected in a range of tick species of the genera *Ixodes*, *Rhipicephalus*, *Hyalomma*, *Amblyomma*, *Haemaphysalis* [204].

The presence of *Wolbachia* in *R. sanguineus* ticks that mainly parasitize dogs has raised a concern as to whether the endosymbionts within the ticks can be transmitted into these animals. Currently, *Wolbachia* is being utilized as a method for vector control in *Aedes* mosquitoes [205,206]. Previous studies on sera of human participants exposed to multiple bites of *Wolbachia*-infected *Aedes* mosquitoes, showed *Wolbachia*-free residues indicating no transmission to humans [207]. On the other hand, an investigation conducted on blood collected from dogs in Haiti found approximately 22% of dogs PCR-positive for *Wolbachia* [208]. Furthermore, *Wolbachia* was detected in blood specimens of dogs and was determined from the filarial nematode *Dirofilaria repens* [209]. *Wolbachia* has also been detected in the blood of cats and it is supposed to be related to the heartworm *Dirofilaria immitis*, which harbors this endosymbiont [210]. Based on these findings, further investigations are required to assess the possibility of *Wolbachia* being transmitted to mammals, including humans, through the feeding of ticks.

## 5. Dermanyssus Gallinae

The genus *Dermanyssus* comprises hematophagous mite species, parasites of birds. The taxonomy of species within the genus was not clearly defined, until now [211]. *Dermanyssus gallinae* (poultry red mite) is very common in layer houses and is considered as the most damaging to laying hens worldwide [212]. The mite belongs to order Mesostigmata and mainly live at all stages in the environment, in cracks or crevices near the hosts’ resting sites, feeding intermittently (every 2–4 days) for short periods (up to 1 h) on the birds during the dark hours [213,214,215]. Its life cycle (from egg to adult) is completed in one to two weeks and takes place through eggs, larvae, two nymphal stages and adults. Especially females of adult and nymphal stages exert hematophagy. This short life cycle, the wide range of optimum temperatures (10–35 °C) and high relative humidity (>70%), usually occurring in egg-laying facilities, contribute to make the mite a pest [216]. Parasite densities can, in fact, reach 50.000–500.000 mites per bird in caged systems [217]. A relationship between the occurrence of mites and hen mortality has been recorded [218].

Although birds are first choice hosts, *D. gallinae* feed on humans and other mammals, too [219], and can act as a vector for several pathogens of poultry [220], as well as zoonotic agents [221]. *D. gallinae*, in fact, feeds on humans too, showing opportunistic feeding habits, in respect to other species within the genus. Immunocompromised people seem to be prone to mites’ attacks and to following pathogens transmission, occurring in more than a third of cases [222].

Poultry red mites spend most of their life in environmental refugia and can survive up to 9 months without feeding [216], so the control should be performed in the environment. The control of *D. gallinae* has been made up by using silicas (in dusts and liquid formulations), exerting a physical action [223,224], yielding satisfying results, when associated with the mechanical cleaning of henhouses.

Chemical acaricides in the environment have been widely applied [215,218,225]. However, a widespread resistance to such molecules has been recorded in the last few decades [218,226,227,228,229,230]; moreover, many acaricides have been withdrawn from the European market [218]. These drugs, in fact, would have a public health impact, occurring as residues in eggs and meat [231,232].

Alternative methods of control have been revised by many authors, encompassing the use of vaccines, pheromones, botanical extracts, natural enemies, acaripathogenic fungi and bacteria, as well as identifying different biological targets for new chemicals [214,215,218,225,233].

### 5.1. Fungi

Entomopathogenic fungi have been assayed to control the mite population. *B. bassiana*, *M. anisopliae, Trichoderma album*, and *P. fumosoroseus* are the most studied fungal species. The use of fungal entomopathogens to control arthropod pests as biological agents would be suggested considering their easy direct penetration through arthropod tegument, the lack of induction of host resistance, the ability to horizontally transmit from fungus-infected to uninfected arthropods, mostly in moist environments [234] and potential damage to flies, lice, and other pests [235,236]. Among the different stages, nymphs show a lower sensitivity to EPFs [237].

*B. bassiana*, *P. fumosoroseus* and *M. anisopliae* were proven to kill several red mites, when administered in high doses, with a variability depending on the isolate [237,238,239,240], being able to cause high mortality within 5 days [238]. The efficacy of *B. bassiana* appeared enhanced, when administered in mixture with *T. album*. These fungi killed up to 80% of treated mites within 10 days [241]. In a more integrated approach, *B. bassiana* showed a synergistic interaction with desiccant dusts (up to 38% higher), maintaining the effectiveness up to 4 weeks [242], with a marked repellent effect [243], and with some essential oils [244]. Problems with the administration of conidia have recently been overcome using corrugated cardboard, infected with high doses of *B. bassiana* spores, acting as an autoinoculation device [245].

Different strains of *M. anisopliae* have been successfully applied to control the mites, under laboratory conditions, demonstrating differences in pathogenicity with a dose- and time-dependent effect [246]. A spray of conidia in sunflower oil applied on field in a poultry farm demonstrated that the amount of conidia should be greater than in laboratory, being difficult to maintain temperature and humidity under control [247], as observed for *M. brunneum* [248]. High temperatures together with low relative humidity negatively affect the efficacy of EPFs, as demonstrated in ticks colonized by *M. anisopliae* [121]. Anyway, *M. anisopliae* was able to reduce the mite population after a week which lasted up 3 weeks [247].

Recently, a native isolate of *Aspergillus oryzae*, previously cultured by a dead *D. gallinae*, was checked for its activity against poultry red mite, showing a lethal activity by the sixth day after the administration of conidial suspension [249].

The main drawbacks for the use of EPFs in the control of *D. gallinae* are related to rapid mite regrowth, the time necessary to allow fungi to grow and low persistence in the environment [250,251], along with the stability of selected strains [252]. For these reasons, genetically modified fungi should be selected.

In conclusion, these entomopathogenic microorganisms seem to show an interesting anti-mite effect against *D. gallinae.* Anyway, considering the complexity of the epidemiology of poultry infection, a multidisciplinary approach would be very advisable [253].

### 5.2. Bacteria

The use of *B. thuringiensis* has been proposed as an alternative control method to chemical acaricides against *D. gallinae* in integrated management programs. It has been observed that *B. thuringiensis* var. *kurstaki* is able to damage the cuticle of *D. galllinae* and cause the loss of mobility of this mite in a period of 24 h [252]. Moreover, Torres and Hernandez [254] observed a moderate mortality of *D. gallinae* from day 2 of application (66%), which increased up to 78% at 7 days at a concentration of 35 mg/mL. Similarly, a previous study by Mullens et al. [255] on the fowl mite *Ornithonyssus sylviarum*, revealed that this mite was susceptible to *B. thuringiensis*, and the authors concluded that the entomopathogen had potential for the development of a control preparation for direct application to poultry.

Microbiota present in *D. gallinae* mites has been studied and four categories of bacteria have been identified: saprophytes, opportunistic pathogens, strict pathogens, and endosymbionts. The last ones are intracellular obligate bacteria able to cause phenotypic and reproductive alterations in their arthropod hosts; they belong to genera *Spiroplasma*, *Cardinium*, *Schineria*, *Rickettsiella* [39,45].

Studies about the presence of *Wolbachia* sp. in *D. gallinae* did not find these bacteria, that are frequently present in other arthropods in which they cause reproductive anomalies [39].

Even though endosymbiotic bacteria living inside *D. gallinae* were found, the effect of these infections on the poultry red mite is not known.

Some studies have been carried out to verify the role of endosymbiotic bacteria living inside *D. gallinae*. Bacteria of the genera *Cardinium*, *Spiroplasma*, *Rickettsiella*, *Schineira* were found in *D. gallinae* sampled from poultry farms located in France and UK [45]. De Luna et al. [45] investigated the endosymbiotic bacteria living inside *D. gallinae* collected from one commercial farm in the UK and different farms in France. Specimens collected in the UK were positive for bacteria of the genera *Cardinium* sp. and *Spiroplasma* sp. From France, seven farms were positive for *Cardinium* sp., one farm was positive for *Spiroplasma* sp., one farm was positive for *Rickettsiella* sp. and two farms were positive for *Schineria* sp. These findings demonstrated that different endosymbionts may be present in *D. gallinae* and the authors supposed that endosymbionts could cause biological modifications to the poultry red mite [43], similarly to what has been observed in other hosts [63].

Based on these observations, it seems that biological control using endosymbiotic bacteria-derived substances that may induce changes to the reproduction of arthropods may be a viable alternative to traditional methods of control of the poultry red mite [45].

## 6. *Psoroptes* sp.

*Psoroptes* mites are non-burrowing Acharina, responsible for ear and body mange of herbivores. *Psoroptes ovis* severely impacts on animal health. It induces an exudative dermatitis in beef cattle and sheep which, when not treated, can lead affected animals to lose condition and, sometimes, to death [256,257]. The parasite of rabbits *Psoroptes cuniculi* (syn *P. ovis* var. *cuniculi*) [258], considered conspecific with *P. ovis* [259], primarily lives on the inner surface of the pinna [260] and is responsible for otoacariasis.

The life cycle of *P. ovis* (egg, larva, two nymphal stages and adults) completely occurs on the host. All parasite stages can pierce the surfaces and feed on tissue fluids. Moreover, the host’s skin produces serous exudate because of a delayed hypersensitivity response induced by allergens from mites’ fecal pellets [256,261].

Psoroptic mange in ovine (sheep scab) and in rabbit hosts (ear cancer) are the most frequently reported clinical forms characterized by severe pruritus. The consequent itching usually distracts the animals from eating, leading to weight loss, and, in sheep, fleece deterioration and reduction in milk and meat production. In rabbit, otherwise, the disease mainly presents as erythema, extreme pruritus, and crusted lesions in the external ear. Sometimes, the mites can spread to other parts of the host’s body, causing generalized scabs in the head, neck, ventral abdomen, and urogenital area [260]. The infections are highly contagious and quickly spread among the animals. *Psoroptes* can survive and maintain their infectivity after 15 days off the host, while mite eggs were able to hatch for up to 7 days in the same conditions [262].

The control of affection and conventional treatment rely on topical organophosphates or injectable formulations of macrocyclic lactones. However, scab mites have developed resistance to conventional acaricide drugs. *Psoroptes* mites have quickly developed resistance to all the synthetic pyrethroids and to propetamphos, without side-resistance to diazinon [263], that nowadays would protect sheep against mites for 8–10 weeks [264]. Injectable macrocyclic lactone formulations are more operator friendly, but, recently, a multiple resistance has been reported [265,266] and the selective pressure of long acting moxidectine would enhance helminthic resistance, too. The use of remedies alternative to ivermectin are welcome, because of several negative aspects relative to this drug. First, ivermectin can induce drug resistance, with the consequent loss of its effectiveness. Moreover, it can be neurotoxic and induce central nervous system depression in treated animals. Subcutaneous treatment is painful for animals and in rabbits it is unsuitable because of their natural behavior; in fact, rabbits usually lick each other in the ears as part of their hygiene, and this aspect may affect the pharmacokinetics of ivermectin, and prolonged treatment could cause intoxication [267,268]. Furthermore, it was demonstrated that the repeated administration of ivermectin subcutaneously in male rabbits causes a decrease in the weight of the sexual organs, which is a negative consequence in the animal production [269]. Finally, the use of ivermectin can represent a threat for people, too, because of the residues in rabbits’ meat for human consumption [270]. Furthermore, acaricide drugs can pass in the environment and in the food chain, occurring as toxic residues in milk and meat, and are banned in organic farms [264].

Alternative biological control can be achieved by using entomopathogenic microorganisms, such as bacteria and molds, or by administering natural compounds [271].

### 6.1. Fungi

Astigmata mites are soft-bodied and have an unsclerotized tegument. This feature would facilitate fungal colonization [272]. Moreover, in diseased animals, the microenvironment of lesions acts as a favorable microclimate for fungal growth. With *Psoroptes* not being a burrowing mite, the parasites live in groups, in strict contact, that allows the direct transmission of mycelia [273].

The rate of parasite killing and thermotolerance are of capital importance to allow the molds to carry out their entomopathogenic activity. These features depend on the selected fungal isolate [274]; *B. bassiana* was reported to show the optimal growth temperature between 25 °C and 28 °C [275], even if some isolates can grow at about 30 °C, with highly reduced activity and may not survive at 34 °C [276].

The first in vitro study on the effects of *B. bassiana* on *Psoroptes* recovered from rabbits [273] stressed a strong lethal activity on both infected adults and on the life span of larvae hatched from infected eggs. Then, an in vitro and in vivo study was performed, demonstrating that *Psoroptes* mites can become infected by entomopathogenic fungi on the skin of sheep, also. These findings showed the feasibility of a direct application of fungal conidia onto the sheep body [277].

In a comparative in vitro study with *Hirsutella thompsonii*, *M. anisopliae* was highly pathogenic and suitable for the control of *P. ovis* [272]. These features were furtherly corroborated by observing the efficiency of the mold in producing fatal infections, as well as the infectiveness of 5-day-old cadavers of mites [278]. *M. anisopliae* shows a higher thermotolerance, when compared with *B. bassiana*, with an optimum 30 °C, but growing at 37.5 °C, also, while no infections were observed at 40 °C [279], confirming the statement “*B. bassiana* and *M. anisopliae* are known to have their optimum of growth at 25 and 30 °C, respectively” [280].

Anyway, although the thermotolerance and virulence of EPFs would depend on the strain, *M. anisopliae* is able to grow up to 35 °C, sharing this feature with *P. farinosus* [274].

The higher infectivity of *M. anisopliae* in comparison with *B. bassiana* was assessed in vivo, too [277]. The strong parasite killing of *M. anisopliae* seems to be related to its ability to induce the oxidative damage of mites [281].

*Scopulariopsis brevicaulis* (teleomorph *Microascus brevicaulis*) is a soilborne ascomycete, occasionally involved as an EPF. The genus has been revised by Sandoval-Denis et al. [282]. The mold showed a dose-dependent pathogenicity for *P.cuniculi* in an in vitro study, being able to colonize the mites, leading them to death. The infected parasites appeared debilitated, lost mobility and quickly died [164]. However, in the same paper, the occurrence and the following cultivation of *Scopulariopsis* sp. from bodies of healthy mites from ear crusts was recorded. This finding would suggest an opportunistic role of this mold versus the tested parasite species. EBFs active versus *D. gallinae*, as well as *Psoroptes* sp. are reported in Table 2.

### 6.2. Bacteria

The in vitro acaricidal effect of *B. thuringiensis* on *P. cuniculi* has been demonstrated. The bacterium can induce histological alterations of this mite, such as the presence of dilated intercellular spaces in the basal membrane, membrane detachment of the peritrophic matrix and morphological alterations in columnar cells of the intestine [283].

The use of mixtures of *B. thuringiensis* with other acaricidal compounds has been proposed. For example, it has been proven that a combination of chitinase and soybean trypsin protease inhibitor effectively suppresses population growth in the flour mite *Acarus siro* [284], and many *B. thuringiensis* strains have chitinolytic activities [285] that could enhance the efficacy in mite control. Lee and coworkers [286] observed that the combination with other natural products such as naphthoquinones induces a decrease in the induction of long-term resistance, with short-term efficacy, and at a low cost.

Similarly, the combined use of *B. thuringiensis* and ivermectin has been proposed by some authors to combat *Psoroptes* sp., in view of a potential synergistic or additive effect with the possibility of lowering the dose of ivermectin [283].

Besides *B. thuringiensis*, that directly acts against *Psoroptes* sp., other bacteria may be involved in the survival of mites. Some studies have been carried out to verify the role of some bacterial strains isolated from mites. *Serratia marcescens* is a Gram-negative bacterium of the family Enterobacteriaceae responsible for infections, including septicemia, in several animal species. It has been proven that this bacterium is pathogenic to several insects, too, including flies and mosquitos with different mechanisms of action [287].

*S. marcescens* has been frequently cultured from *Psoroptes* sp. mites, but it is not clear if the bacterium acts as endosymbiont or has anti-mite activity. Perrucci and coworkers [288] observed that *P. cuniculi* does not need *S. marcescens* to live and infect healthy rabbits. However, the authors found that only rabbits infested with *S. marcescens*-free *P. cuniculi* mites presented crusts in their ears, whereas mites and/or eggs were only detected in the ear cerumen of all rabbits infested with *S. marcescens*-infected mites.

Table 3 summarizes EPBs active against *Psoroptes* sp, *D. gallinae* and some tick species.

## 7. *Varroa destructor*

*Varroa destructor* is a parasite Mesostigmata mite, exerting a huge impact on beekeeping. It has become a global parasite, switching host onto *Apis mellifera* from *Apis cerana*. Varroasis is often a threat for colonies, when nearby colonies collapse [289]. Without a treatment, an infected colony dies within 2 years post infection [290]. The life cycle consists of a phoretic phase when adult hosts carry mites within and between colonies and a reproductive phase, when *Varroa* lays eggs inside the bee brood cells [291]. Mature female daughters are produced in worker brood cells and daughters in drone cells and remain immobile until prepupae are present. Then they climb on them and create a feeding site by puncturing the host’s cuticle and feed on the larval fat body, parthenogenic mites develop, then oviposition starts. When bees emerge from the cells, they have mites feeding on them [292]. The affected hosts show weight loss, with a deficit in reproductive fitness. The mites would prefer nurse bees [293], and modify bees’ behavior, are able to mimic a host’s cuticular hydrocarbons to escape the hygienic behavior of the host [294] and quickly shift to acaricide resistance. *Varroa* can transmit deformed wing virus and acute bee paralysis virus. Noel et al. [291] have recently reviewed the main control options. Chemical control is based on conventional miticide products acting on *Varroa* on adult bees or, when administered in strips, acting on mites emerging from the brood cells. However, these drugs leave residues in hive products, and resistance to acaricide is increasing. Organic acids or terpenes such as thymol are used in organic control, but they would decrease worker population, increasing capping brood removal or decreasing sperm quality. Lithium chloride appears as a selective inhibitor of *Varroa* acetylcholinesterase, such as the use of predators (with interesting laboratory results, but not on colony). Promising is the use of RNA interference to knock down specific genes of *Varroa*, although still experimental. *V. destructor* has been reported to be susceptible to the entomopathogenic fungi, *M. anisopliae*, *B. bassiana*, *Verticillium lecanii*, *Hirsutella* spp. [3,295], *Hirsutella thompsonii* [296,297], *B. bassiana* [298] and *M. anisopliae* [299,300]. *Clonostachys rosea* (formerly *Gliocladium roseum*) is an Ascomycete, belonging to Hypocreales, widely distributed in soil, and provided by an endophytic ability in tissues from several plants. The mold produces conidia and chlamydospores. Colonies on potato dextrose agar are greyish white when grown in the dark, while appearing yellow to orange under lighter conditions [301]. *C. rosea* was able to kill 60% of mites, in comparison with *B. bassiana* and *M. anisopliae*, which caused the death of 90% of mites. All the acaripathogenic molds were reported to be able to control *V. destructor* by preventing the gene suppression of bee immunity, induced by the mite, too [302]. However, the main shortcoming for the use of acaripathogenic fungi in beekeeping is due to the potential pathogenicity of these fungi for insects, too.

Alquisira-Ramirez et al. [303] observed that *B. thuringiensis* could be an effective alternative to control *V. destructor*, because the bacterium is virulent to the mite but does not cause mortality in bees. In fact, no toxic effects of the proteins of *B. thuringiensis* have been demonstrated for the larvae and adults of *A. mellifera*, maybe because the pH of the bee intestine is usually acidic, whereas *B. thuringiensis* toxins are activated at alkaline pH values [304].

## 8. Zoonotic Potential of EPFs

A potential zoonotic activity of EPFs has been reported. *M. robertsii*, *M. guizhouense*, *M. brunneum* and *M. pingshaense* (specie complex *M. anisopliae*) were referred as the species involved with human infection, mostly keratitis [305], although two cases of keratitis due to *M. anisopliae* have been reported in soft contact lens wearers [306]. However, considering a last further recent taxonomic study, comprising the description on new species [20], within the known complexes, it is very hard to state the lack of zoonotic ability of *Metarhizium* species, used as mycoacaricide. On the other hand, *B. bassiana* was identified as responsible for mycotic keratitis in a patient involved in occasional agriculture work [307] and, interestingly, in the same study, 14 clinical cases of *B. bassiana* keratitis were revised. Five out of the twelve patients with anamnestic data were working in agriculture.

*Bacillus thuringiensis* has been associated to different human infections; it has been cultured from marginal and apical periodontitis, wounds, corneal ulcera and gastrointestinal infections in humans [308]. Even though this EPB is not considered as a traditional zoonotic agent, its presence in different forms of human infections suggests that, at least in immunocompromised patients, it could represent a risk. *B. thuringiensis*, similarly to *B. cereus*, produces several virulence factors potentially acting against mammalian cells, such as hemolysins and enterotoxins [309].

For these reasons, the use of *B. thuringiensis* in pest control should be carried out with attention to avoid possible infections, mainly in operators.

*Bacillus cereus* has been involved in human periodontitis, too [308], as well as in other human infections, mainly of the gastrointestinal tract [310]. *B. cereus* is thus a well-known pathogen for humans and animals and for this reason, its use is not recommended in pest control.

## 9. Conclusions

The control of ectoparasites requests the development of novel strategies and, among them, the use of entomopathogenic microorganisms appears as a promising tool to achieve an eco-friendly approach. Several studies have been accomplished, both in crops’ defense and in sanitary entomology, mostly in fighting mosquitoes. The present study has revised the literature dealing with the application of these organisms to manage some veterinary parasitosis, caused by Acari.

Most of data refer to ticks’ control, showing the feasibility of the environmental application of this strategy, as well as important differences in the sensitivity of ticks and pathogenicity of EPFs, that make a preliminary laboratory assay mandatory.

Entomopathogenic microorganisms appear as important for their environmental sustainability, for the lack of resistance induction in parasites and, in general, for their safety towards hosts, proving the ability to break the life cycle of both these pests and of several vector-borne agents, zoonotic, also, in a One Health perspective.

These tools appear promising in an integrate approach, too, and their administration with conventional acaricide drugs or, in a green approach, with different plant extracts is advisable. Finally, the management of *D. gallinae* should be considered as an ideal candidate for an in-field application of this strategy, considering the withdrawal of several conventional acaricide from the market. Further study and in-field research are needed to improve a large-scale application, considering the possible impact on non-target species, too.

## Figures and Tables

**Table 1:** Entomopathogenic fungi (EPFs) active versus different tick species.

Tick Species	EPFs	References
*Amblyomma americanum*	*Beauveria bassiana*	[149]
*Amblyomma parvum*	*Metarhizium anisopliae*	[136]
*Amblyomma variegatum*	*Beauveria bassiana*	[152]
*Amblyomma variegatum*	*Metarhizium anisopliae*	[152]
*Amblyomma variegatum*	*M. anisopliae + B. bassiana*	[153]
*Boophilus microplus*	*Beauveria bassiana*	[186]
*Boophilus microplus*	*Metarhizium anisopliae*	[130,131,186,187,188,189]
*Boophilus* sp.	*Fusarium* sp. *Metarhizium anisopliae*	[3]
*Dermacentor albipictus*	*Beauveria bassiana*	[138]
*Dermacentor albipictus*	*Metarhizium anisopliae*	[138]
*Dermacentor albipictus*	*Metarhizium brunneum*	[138]
*Dermacentor marginatus*	*Aspergillus fumigatus*	[190]
*Dermacentor marginatus*	*Trichothecium roseum*	[191]
*Dermacentor reticulatus*	*Isaria fumosorosea*	[139]
*Dermacentor reticulatus*	*Beauveria bassiana*	[139]
*Dermacentor reticulatus*	*Metarhizium anisopliae*	[139]
*Dermacentor reticulatus*	*Metarhizium robertsii*	[139]
*Dermacentor* sp.	*Beauveria bassiana*	[107]
*Dermacentor variabilis*	*Metarhizium anisopliae*	[116]
*Dermacentor variabilis*	*Beauveria bassiana*	[116]
*Dermacentor variabilis*	*Scopulariopsis brevicaulis*	[167]
*Haemaphysalis longicornis*	*Beauveria bassiana*	[142]
*Haemaphysalis qinghaiensis*	*Metarhizium anisopliae*	[137]
*Haemaphysalis qinghaiensis*	*Beauveria bassiana*	[137]
*Hyalomma anatolicum*	*Beauveria bassiana*	[140]
*Hyalomma anatolicum*	*Metarhizium anisopliae*	[140]
*Hyalomma anatolicum*	*Paecilomyces lilacinus*	[140]
*Hyalomma lusitanicum*	*Beauveria bassiana*	[147,148]
*Hyalomma scupense*	*Aspergillus fumigatus*	[190]
*Ixodes dammini*	*Aspergillus ochraceus*	[3]
*Ixodes dammini*	*Metarhizium anisopliae*	[192]
*Ixodes ricinus*	*Conidiobolus coronatus*	[108]
*Ixodes ricinus*	*Aspergillus flavus*	[106]
*Ixodes ricinus*	*Aspergillus fumigatus*	[106]
*Ixodes ricinus*	*Aspergillus niger*	[107]
*Ixodes ricinus*	*Aspergillus parasiticus*	[107]
*Ixodes ricinus*	*Beauveria bassiana*	[3,139]
*Ixodes ricinus*	*Beauveria brognardi*	[108]
*Ixodes ricinus*	*Paecilomyces farinosus*	[108]
*Ixodes ricinus*	*Paecilomyces fumosoroseus*	[107,108]
*Ixodes ricinus*	*Penicillium insectivorum*	[106]
*Ixodes ricinus*	*Trichothecium roseum*	[191]
*Ixodes ricinus*	*Verticillium aranearum*	[108]
*Ixodes ricinus*	*Verticillium lecanii*	[107,108]
*Ixodes ricinus*	*Metarhizium anisopliae*	[139]
*Ixodes ricinus*	*Metarhizium robertsii*	[139]
*Ixodes ricinus*	*Isaria fumosorosea*	[139]
*Ixodes scapularis*	*Metarhizium brunneum*	[116,117,118,143]
*Ixodes scapularis*	*Metarhizium anisopliae*	[116]
*Ixodes scapularis*	*Beauveria bassiana*	[116]
*Rhipicephalus annulatus*	*Metarhizium brunneum*	[144]
*Rhipicephalus appendiculatus*	*Aspergillus* sp.	[193]
*Rhipicephalus appendiculatus*	*Fusarium* sp.	[193]
*Rhipicephalus appendiculatus*	*Metarhizium anisopliae*	[151,152,193]
*Rhipicephalus appendiculatus*	*Beauveria bassiana*	[151]
*Rhipicephalus appendiculatus*	*M. anisopliae + B. bassiana*	[152]
*Rhipicephalus decoloratus*	*Beauveria bassiana*	[150]
*Rhipicephalus microplus*	*Metarhizium robertsii*	[126,127,132,135]
*Rhipicephalus microplus*	*Beauveria bassiana*	[129,132,145,146,155,158,159,160]
*Rhipicephalus microplus*	*Metarhizium anisopliae*	[129,132,133,155,158,159,160]
*Rhipicephalus microplus*	*Paecilomyces lilacinus*	[129]
*Rhipicephalus microplus*	*Isaria fumosorosea*	[162]
*Rhipicephalus microplus*	*Isaria farinosa*	[162]
*Rhipicephalus microplus*	*Purpurocillium lilacinus*	[162]
*Rhipicephalus sanguineus*	*Aspergillus ochraceus*	[109]
*Rhipicephalus sanguineus*	*Fusarium* sp.	[194]
*Rhipicephalus sanguineus*	*Curvularia lunata*	[110]
*Rhipicephalus sanguineus*	*Rhizopus thailandensis*	[110]
*Rhipicephalus sanguineus*	*Rhizopus arrhizus*	[110]
*Rhipicephalus sanguineus*	*Metarhizium anisopliae*	[113,114,115,116]
*Rhipicephalus sanguineus*	*Metarhizium flavoviride*	[114]
*Rhipicephalus sanguineus*	*Isaria fumosorosea*	[114]
*Rhipicephalus sanguineus*	*Beauveria bassiana*	[116]

**Table 2:** Entomopathogenic fungi (EPFs) active versus different mite species.

Mite Species	EPFs	References
*Dermanyssus gallinae*	*Beauveria bassiana*	[237,240,243,245]
*Dermanyssus gallinae*	*B. bassiana + Trichoderma album*	[241]
*Dermanyssus gallinae*	*Metarhizium anisopliae*	[246,247]
*Dermanyssus gallinae*	*Metarhizium brunneum*	[248]
*Dermanyssus gallinae*	*Aspergillus oryzae*	[249]
*Psoroptes ovis*	*Beauveria bassiana*	[269,277]
*Psoroptes ovis*	*Hirsutella thompsonii*	[272]
*Psoroptes ovis*	*Metarhizium anisopliae*	[272,277]
*Psoroptes cuniculi*	*Scopulariopsis* sp.	[164]

**Table 3:** Entomopathogenic bacteria (EPBs) active against different arthropod species.

Arthropod Species	EPBs	References
*Argas persicus*	*Bacillus thuringiensis*	[197]
*Dermacentor andersoni*	*Proteus mirabilis*	[201]
*Dermacentor reticulatus*	*Bacillus thuringiensis*	[199]
*Hyalomma dromedarii*	*Bacillus thuringiensis*	[197,200]
*Ixodes ricinus*	*Bacillus thuringiensis*	[199]
*Ixodes scapularis*	*Bacillus thuringiensis*	[198]
*Rhipicephalus microplus*	*Bacillus thuringiensis*	[195]
*Dermanyssus gallinae*	*Bacillus thuringiensis*	[254]
*Ornithonyssus sylviarum*	*Bacillus thuringiensis*	[255]
*Psoroptes* sp.	*Bacillus thuringiensis*	[283]
